# Left Ventricular Diastolic Function: Comparison of Slow Coronary Flow Phenomenon and Left Ventricular Hypertrophy in the Absence of Obstructive Coronary Disease

**DOI:** 10.7759/cureus.24789

**Published:** 2022-05-06

**Authors:** Niya E Semerdzhieva, Stefan V Denchev, Mariana V Gospodinova

**Affiliations:** 1 Emergency Department, National Heart Hospital, Sofia, BGR; 2 Cardiology Department, Medical Center 'Mediva', Sofia, BGR; 3 Cardiology Department, University Hospital 'St Ivan Rilski', Sofia, BGR

**Keywords:** left ventricular filling pressure, left ventricular relaxation, corrected thrombolysis in myocardial infarction frame count, slow coronary flow phenomenon, left ventricular hypertrophy

## Abstract

Background

An interplay of myocardial structural abnormalities and coronary arterial dysfunction underlies the worsening left ventricular compliance. The conventionally used angina drugs have demonstrated a beneficial effect on both angina and coronary flow in cases with microvascular dysfunction and non-obstructive coronary disease. Despite that, vasoactive therapy only partially affects diastolic function in this patient population.

Purpose

This retrospective study was planned to evaluate the association of myocardial mass, delayed epicardial coronary flow, and vasoactive drugs with parameters of diastolic function in two cohorts with preserved left ventricular function and non-obstructive coronary disease in patients with slow coronary flow phenomenon (SCFP) and patients with the hypertensive disease and left ventricular hypertrophy.

Material and methods

The epicardial coronary flow was evaluated in 48 patients with unstable angina in the absence of coronary stenosis >50%, by applying the methods of corrected thrombolysis in myocardial infraction frame count (cTFC). The abnormalities in the left ventricular function were assessed by echocardiography using PW-Doppler of the diastolic mitral inflow and tissue Doppler imaging. Twenty-one (43.8%) patients were diagnosed with SCFP, and twelve patients (25%) had slow epicardial coronary flow, hypertensive disease, and ventricular hypertrophy (SF_LVH_). The remaining 15 (31.3%) were patients with ventricular hypertrophy, hypertension, and non-delayed epicardial coronary flow (NF_LVH_).

Results

The patients with SF_LVH_ showed reduced peak early diastolic lateral mitral annular velocity (e’_L_) when compared to SCFP (7.1±1.9cm/s vs 8.6±2.1 cm/s, p=0.045) and NF_LVH_ (7.1±1.9 cm/s vs 8.7±1.8 cm/s, p=0.018). A borderline significant difference was observed for the peak early diastolic septal mitral annular velocity (e’S) between the patients with SF_LVH_ and SCFP ( 7.0±1.3 cm/s vs 8.3±2.1 cm/s, p=0.057). The ratio of mitral diastolic inflow velocity to early diastolic velocity of the mitral annulus (E/e’) in the SF_LVH_ group was a tendency higher than E/e’ of the patients with SCFP (9.8±3.1 vs. 8.2±2.1, p=0.084) and NF_LVH _(9.8±3.1 vs. 7.8±1.5, p=0.051) .In the group with left ventricular hypertrophy, E/e’ >10 was more frequently observed in patients with a marked delay in the epicardial flow (33.1 ± 13.1 frames vs. 25.4 ± 11.8 frames, p=0.011) and higher left ventricular mass (146.9 ± 17.7 g/m2 vs. 126.1 ± 121.5 g/m2, p=0.027).

Conclusions

Patients with microvascular angina represent a diverse population. The echocardiographic parameters of left ventricular relaxation (e') and end-diastolic pressure (E/e’) are abnormally altered in the population with left ventricular hypertrophy compared to SCFP. The delayed epicardial flow further impairs diastolic function in hypertensive patients with hypertrophy and non-obstructive coronary disease.

## Introduction

Studies in cohorts with heart failure with preserved ejection fraction (HFpEF) in the absence of coronary atherosclerosis have observed a stiffer left ventricle and reduced longitudinal systolic function in patients with pronounced epicardial coronary flow abnormalities [1,2]. In addition, the results of prior experimental and animal models have indicated that an interplay of myocardial structural abnormalities and coronary arterial dysfunction underlied the worsening left ventricular compliance [3].

Major classes of conventionally used angina drugs have demonstrated beneficial effects on ischemia and coronary flow in cases with microvascular dysfunction and non-obstructive coronary disease. Nevertheless, the medications only partially affect diastolic function in this patient population [4-7].

Thus, this retrospective study was designed to evaluate the association of myocardial mass, delayed epicardial coronary flow, and vasoactive drugs with parameters of diastolic function in two cohorts with preserved left ventricular function and non-obstructive coronary disease. Specifically, the two cohorts included patients with slow coronary flow phenomenon (SCFP) and patients with hypertensive disease and left ventricular hypertrophy.

## Materials and methods

Тhis research consisted of a single-center, cohort, retrospective study. The study group consisted of 48 patients. Only patients diagnosed with non-obstructive coronary disease (defined as the absence of coronary stenosis of >50% at the time of quantitative coronary angiography) were included in the study. The patients were admitted with unstable angina to the University Hospital 'Alexandrovska’ in Sofia between 2006 and 2008. Thirty-nine patients (70%) underwent symptom-limited exercise stress electrocardiographic tests on the modified Bruce protocol in this clinic prior to angiography [8]. For the remainder of the group, the angiographic study was performed because of angina refractory to optimal anti-ischemic treatment. Twenty-one (43.8%) patients were diagnosed with SCFP, and twelve patients (25%) had slow epicardial coronary flow and ventricular hypertrophy associated with hypertension. Furthermore, fifteen patients (31.3%) had ventricular hypertrophy subsequent to hypertension and normal epicardial coronary flow. The patients with coronary stenoses no greater than 40% and without LVH comprised the SCFP group. Hypertension was defined as clinically measured systolic blood pressure (BP) values ≥140 mmHg and/or diastolic BP values ≥90 mmHg [9]. The diagnosis of hypertension was based on the results of prior multiple BP measurements, which were taken on separate occasions over a period of time and included at least three visits and at least two BP measurements per visit [10]. The diagnosis was made after BP assessment over a maximal period of several months for patients with only slightly elevated BP with large spontaneous variations in BP [10]. Both patients with only clinically measured hypertension and only home-based BP elevation were also diagnosed as hypertensive patients. The diagnostic procedures also included medical history for systemic hypertension controlled by the intake of BP-lowering drugs and instrumental investigations (continuous 24-hour ambulatory BP monitoring).

Epicardial coronary flow was evaluated by using the corrected thrombolysis in myocardial infarction (TIMI) frame count (cTFC) method [11]. This method represented a quantitative measurement of epicardial coronary flow velocity. Specifically, it was defined by the number of cineframes required for contrast to first obtain standardized distal coronary landmarks [11]. The following distal landmark branches were used for the analysis: the distal bifurcation of the left anterior descending branch (LAD); in the circumflex system, the distal bifurcation of the segment with the longest total distance; and in the right coronary artery (RCA), the first branch of the posterolateral artery [11]. This discrepancy in the length of the LAD and the other coronary arteries was corrected or adjusted by dividing the TIMI frame count of the LAD by a factor of 1.7 [11].

All of the patients underwent standard echocardiography (including 2D, M-mode, spectral, color Doppler techniques, and tissue Doppler imaging [TDI]). The echocardiography was performed in accordance with the recommendations of the American Society of Echocardiography and the European Association of Cardiovascular Imaging [12,13]. The LV ejection fraction (EF) was measured using the Simpson method. Values of LVEF lower than 50% were the sole criterion for defining left ventricular systolic dysfunction.

The following diastolic function indices were determined: early (E-wave) and late (A-wave) maximal rates of the left ventricular diastolic filling (diastolic mitral valve inflow); the time of attenuation of the E-wave (deceleration time [DT]); and peak early diastolic mitral annular septal (e’s) and lateral (e’L) velocities. Left ventricular hypertrophy (LVH) was defined as a thickness of the ventricular septum (IVS) or the posterior wall (LVPW) of the left ventricle ≥12 mm on echocardiography, as well as myocardial mass greater than 95 g/m2 for the female patients and greater than 115 g/m2 for the male patients. The myocardial mass was calculated by using the linear method:

0.8x1.04 х [(EDD-IVS-LVPW)3 - EDD3]+0.6 grams;

EDD was the dimension of the left ventricle at end-diastole. The myocardial mass of each patient was divided by the body surface area. In our study report, we used the phrases ‘left ventricular hypertrophy subsequent to systemic hypertension' or ‘left ventricular hypertrophy related to systemic hypertension’ to address this specific alteration in heart structure as part of the complex pathologies that are induced by systemic hypertension and included the term ‘hypertensive heart disease.’

The exclusion criteria for the study included previous coronary revascularization procedures, thrombolytic therapy for myocardial infarction, left ventricular systolic dysfunction (LVEF <50%), wall motion abnormalities at rest, cardiomyopathy, coronary aneurysm, ectasia and fistula, valve disease, acute or chronic inflammatory disease, recent fracture or surgical procedures, and any type of shock and neoplastic disease. In addition, cases with suboptimal angiographic or echocardiographic imaging study results were also excluded from the study.

All of the patients signed written informed consent forms for all diagnostic tests. This retrospective study was approved by the Ethics Committee of University Hospital ‘Alexandrovska’ (incoming No of approval: 298/Oct 31, 2018) and complied with the Declaration of Helsinki.

The analysis of the data was performed by using SPSS version 19.0 (IBM Corp., Armonk, NY, USA). Categorical variables were presented as counts and percentages, and continuous variables were presented as the mean and SD. Categorical variables were analyzed via the chi-squared test or Fisher’s exact test. The normality of the continuous variables was tested by using the Kolmogorov-Smirnov and Shapiro-Wilk tests. Depending on the results of Levene’s test, the variables with a normal distribution were compared with the Student’s t-test or the Welch test. The nonparametric variables were analyzed with the Mann-Whitney test. One-way ANOVA was applied to evaluate the difference between three continuous variables. Associations with α <0.05 were considered to be statistically significant.

## Results

The three groups did not significantly differ with respect to cardiovascular risk profile, systolic function parameters, or the type of vasoactive therapy that was used. The patients with SF_LVH_ and NF_LVH_ more frequently used (although insignificantly) diuretics and less frequently used antiplatelet therapy than those with SCFP (Table 1).

**Table 1:** Clinical profile and echocardiographic, angiographic data. SCFP: Slow coronary flow phenomenon; SF_LVH: _Left ventricular hypertrophy and slow coronary flow secondary to hypertension; NF_ LVH: _Left ventricular hypertrophy and non-delayed coronary flow; DM: Diabetes mellitus; EDVI, ESVI: Indices of end-diastolic and end-systolic volume; EF: Ejection fraction; IVS: Thickness of ventricular septum; LVPW: Thickness of left ventricular posterior wall; ACE-I/ARB: Angiotensin-converting enzyme inhibitor/Receptor antagonist.

Patients	SCFP n, %	SF_LVH _ n,%	NF_LVH_ n,%	P-value
Age	56.3±8.9	59.1±8.5	57.5±9	0.704
Men	10 (47.6%)	4 (33.3%)	1 (6.7%)	NS
Women	11 (52.4%)	8 (66.7%)	14 (93.3%)	NS
Hypertension	17 (81%)	12 (100%)	15 (100%)	NS
Dyslipidemia	17 (81%)	12 (100%)	10 (80%)	NS
Diabetes mellitus	3 (14.3%)	6 (50%)	3 (20%)	NS
Smoking	3 (14.3%)	1 (8.3%)	4 (26.4%)	NS
BMI, kg/m^2^	28.5±3.7	29.5±3.7	28.7±3.2	0.752
EDVI, ml/m^2^	62±14	62±15	72±20	0.144
ESVI, ml/m^2^	19±4	20±7	23±7	0.110
EF, %	68±4	71±7	68±7	0.352
IVS, mm	10.4±0.8	12.4±1.1	12.4±1.8	<0.0001
LVPW, mm	10.3±0.9	12±1	12.2±1.4	<0.0001
Positive exercise ECG	9 (56.3%)	5 (50%)	8 (61.5%)	NS
β-blocker	11 (55%)	4 (33.3%)	6 (40%)	
β-blocker+CCB±nitrate	5 (25%)	5 (41.7%)	7 (46.7%)	NS
β-blocker+nitrate	4 (20%)	3 (25%)	2 (13.3%)	
АCE-I/ARB	11 (52.4%)	11 (91.7%)	14 (93.3%)	NS
Statin	14 (66.7%)	6 (50%)	8 (53.3%)	NS
Aspirin/Clopidogrel	20 (95%)	12 (100%)	11 (73.3%)	0.042
Diuretics	4 (19%)	7 (58.3%)	10 (66.7%)	NS

**Figure 1:**
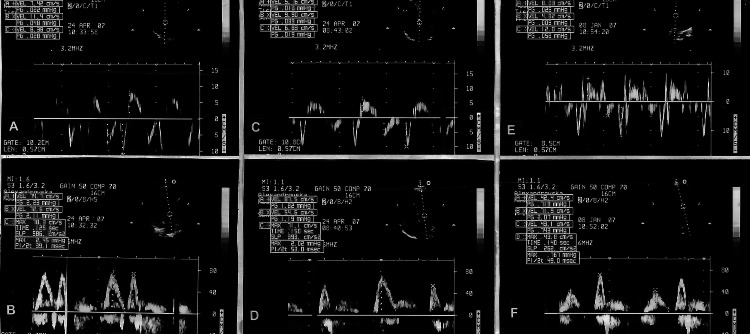
Peak early septal diastolic velocity of mitral annulus: e's and ratio of the peak early diastolic mitral valve inflow velocity to peak early septal diastolic velocity of mitral annulus: E/e's. A-B:* *Patient with SCFP: e'_s_: 7.4 cm/s; E: 75 cm/s; E/e'_s_: 10.6; C-D: Patient with NF_LVH_: e'_s_: 5.8 cm/s; E: 67 cm/s; E/e'_s_: 10.1; E-F*:* Patient with SF_LVH_: e'_s_: 4.6 cm/s; E: 72 cm/s; E/e'_s_: 15.7.

The patients with LV hypertrophy and delayed epicardial flow showed reduced lateral early diastolic mitral annular velocity (e’L) when compared to SCFP and NF_LVH_. A borderline significant difference was observed for the septal early diastolic mitral annular velocity (e’s) of SF_LVH_ and SCFP. Moreover, the filling pressures (E/e’) in the SF_LVH_ tended to be higher than the E/e’ of the patients with SCFP and NF_LVH_ (Figure 1, Tables 2-3).

**Table 2:** Indices of diastolic function and coronary flow: difference of SCFP and SFLVH. Depic: Epicardial coronary diameter; cTFC: Corrected TIMI frame count; E- and A-wave: Peak early and late rates of diastolic left ventricular filling; DT: Time of decrease in early diastolic left ventricular filling; e'_L _and e'_S: _Peak early diastolic lateral and septal mitral annular velocities; E/e’: Ratio of early diastolic mitral inflow velocity to early diastolic mitral annular velocity.

Patients	SCFP	SF_LVH _	P-value
Depic mm	3.7±1.0	4.1±0.8	0.137
cTFC frames	36.2±13.4	37.9±11.9	0.447
E сm/s	75.7±30.0	64.3±12.8	0.267
A сm/s	54.1±20.5	54.5±21.4	0.835
DT msec	190.1±41	182.7±30	0.815
E/A	1.2±0.8	1.8±1.6	0.358
e’_L _cm/s	8.6±2.1	7.1±1.9	0.045
e’s cm/s	8.3±2.1	7.0±1.3	0.057
E/e’	8.2±2.1	9.8±3.1	0.084
Myocardial mass, gr/m^2^	89.5±13.2	127.2±21.3	<0.0001

**Table 3:** Indices of diastolic function in patients with hypertrophy with and without delayed coronary flow. SF_LVH_: Slow coronary flow associated with left ventricular hypertrophy secondary to hypertension; NF_LVH_: Patients with left ventricular hypertrophy and non-delayed coronary flow; Depic: Epicardial coronary diameter; cTFC: Corrected TIMI frame count; E-wave and A-wave: Peak early and late rates of diastolic left ventricular filling; DT: Time of decrease in early diastolic left ventricular filling; e'_L _and e'_S_:Peak early diastolic lateral and septal velocities of the mitral annulus; E/e’: Ratio of early diastolic mitral inflow velocity to early diastolic velocity of the mitral annulus.

Patients	SF_LVH _	NF_LVH_	P-value
Depic, mm	4.1±0.8	3.3±0.7	0.057
cTFC, frames	37.9±11.7	20±4.1	<0.0001
E, cm/s	64.3±12.8	59.1±12.6	0.274
A, cm/s	54.5±21.4	56.4±20	0.814
DT, msec	182.7±30.4	180.7±36.5	0.884
E/A	1.8±1.6	1.3±1.0	0.695
e’_L __, _cm/s	7.1±1.9	8.7±1.8	0.018
e’s, cm/s	7.0±1.3	7.7±1.8	0.148
E/e’	9.8±3.1	7.8±1.5	0.051
Myocardial mass, gr/m^2^	127.2±21.3	133.2±24.4	0.506

Increased early mitral inflow velocity and reduced longitudinal septal function (e's) corresponded to the presence of left ventricular hypertrophy in the entire non-obstructive disease cohort (Table 4).

**Table 4:** Mitral inflow and tissue Doppler echocardiography in diastole patients with SCFP compared to patients with left ventricular hypertrophy. Depic: Epicardial coronary diameter; cTFC: Corrected TIMI frame count; E- and A-wave: Peak early and late rates of diastolic left ventricular filling; DT: Time of decrease in early diastolic left ventricular filling; e'L and e'S: Peak early diastolic lateral and septal mitral annular velocities; E/e’: Ratio of early diastolic mitral inflow velocity to early diastolic mitral annular velocity.

Patients	SCFP (n=21)	LVH (n=27)	P-value
Depic, mm	3.7±1.0	3.7±0.8	0.906
cTFC, frames	36.2±12.3	28±12.3	0.025
E, cm/s	74.7±30	61.4±12.2	0.042
A, cm/s	56.1±20.5	55.5±20.2	0.926
DT, msec	190.1±41	181.6±33.3	0.444
E/A	1.3±0.8	1.5±1.3	0.714
e’_L __, _cm/s	8.6±2.1	8.0±2.0	0.284
e’s, cm/s	8.3±2.1	7.4±1.6	0.079
E/e’	8.2±2.1	8.7±2.6	0.501
Myocardial mass, gr/m^2^	89.5±13.2	130.6±22.9	<0.0001

Diastolic function parameters were similar in the slow coronary flow patients and those with left ventricular hypertrophy with the normal flow (Table 5).

**Table 5:** Diastolic function parameters in patients with delayed epicardial coronary flow with and without hypertrophy: a comparison with left ventricular hypertrophy and normal flow. Depic: Epicardial coronary diameter; cTFC: Corrected TIMI frame count; E- and A-wave: Peak early and late rates of diastolic left ventricular filling; DT: Time of decrease in early diastolic left ventricular filling; e'L and e'S: Peak early diastolic lateral and septal mitral annular velocities; E/e’: Ratio of early diastolic mitral inflow velocity to early diastolic mitral annular velocity.

Patients	SCFP+SF_LVH _ (n=27)	NF_LVH_ (n=15)	P-value
Depic, mm	3.8±1.0	3.4±0.7	0.090
cTFC, frames	36.8±12	20±4.1	<0.0001
E, cm/s	70.9±25.4	59.1±11.6	0.092
A, cm/s	55.5±20.5	56.4±20	0.887
DT, msec	187.5±87.3	180.7±36.5	0.569
E/A	1.5±1.2	1.3±1.0	0.688
e’_L __, _cm/s	8.1±2.1	8.7±1.8	0.331
e’s, cm/s	7.9±1.9	7.7±1.8	0.456
E/e’	8.8±2.6	7.8±1.5	0..153
Myocardial mass, gr/m^2^	103.2±24.6	133.2±24.4	<0.0001

In the group with SCFP and the entire study group, E/e’ in the highest tertile (E/e’>10) was not dependent on either coronary flow velocity or myocardial mass. In the presence of left ventricular hypertrophy, E/e’>10 was more frequently observed in patients with a marked delay in epicardial flow and higher left ventricular mass. In the group with delayed epicardial flow (including SCFP and patients with slow flow in the presence of LVH), E/e’ was significantly related only to the elevated myocardial mass (Table 6).

**Table 6:** E/е’ relation with the myocardial mass and coronary flow. SCFP: Slow coronary flow phenomenon; SF_LVH_: Slow coronary flow associated with left ventricular hypertrophy secondary to hypertension; NF_LVH_: Patients with left ventricular hypertrophy and non-delayed coronary flow; Depic: Epicardial coronary diameter; cTFC: Corrected TIMI frame count.

SCFP	E/e’<10 (n=14)	E/e’>10 (n=7)	P-value
Depic, mm	3.7±1.2	3.5±0.6	0.574
cTFC, frames	36.7±11.9	35±14.4	0.787
Myocardial mass, gr/m^2^	86.1±19.4	97.8±14.9	0.064
LVH (SF_LVH_+NF_LVH_)	E/e’<10 (n=19)	E/e’>10 (n=8)	P-value
Depic, mm	3.6±0.8	3.8±0.9	0.640
cTFC, frames	25.4±11.8	33.1±13.1	0.011
Myocardial mass, gr/m^2^	126.1±21.5	146.9±17.7	0.027
SCFP + SF_LVH_	E/e’<10 (n=21)	E/e’>10 (n=12)	P-value
Depic, mm	3.9±1.1	3.8±0.8	0.800
cTFC, frames	37.1±11.9	36.4±12.5	0.887
Myocardial mass, gr/m^2^	95.3±18.4	116.9±28.6	0.031
All patients	E/e’<10 (n=33)	E/e’>10 (n=15)	P-value
Depic, mm	3.7±1.0	3.7±0.8	0.983
cTFC, frames	30.3±12.7	35±13	0.262
Myocardial mass, gr/m^2^	109.7±27.3	120.2 ± 29.8	0.257

The majority of patients were using bisoprolol or metoprolol (92.9%) as monotherapy or combined with one of the following calcium channel blockers: amlodipine, felodipine, or lercanidipine (97.6%). None of the patients used first-generation dihydropyridines (e.g., nifedipine). One patient was on therapy with oral verapamil hydrochloride; the other two patients were on therapy with a first-generation β-blocker (atenolol). The β-blocker of the third generation (nebivolol) was included in the treatment regimen along with the CCB of one participant. The intake of diuretics was not associated with a lower preload (Е/e' no diuretics vs. Е/e' diuretics 8.7±2.5 [n=27] vs. 8.2±2.2 [n=21], p=0.557). The difference in diastolic function parameters in relation to the vasoactive therapy used in the groups with SCFP and patients with LVH could not be discussed due to the small sample size. However, the early-diastolic septal and lateral mitral annular velocities seemed to be substantially reduced. E/e’ was higher in the patients with LVH on therapy with β-blocker with nitrate compared to these variables in the LVH subgroup taking β-blocker alone or combined with a calcium blocker (Tables 7-8).

**Table 7:** Vasoactive therapy and indices of diastolic function in the group with SCFP. SCFP: Slow coronary flow phenomenon; Depic: Epicardial coronary diameter; cTFC: Corrected TIMI frame count; E-wave and A-wave: Peak early and late rates of diastolic left ventricular filling; DT: Time of decrease in early diastolic left ventricular filling; e'_L_ and e'_S_: Peak early diastolic lateral and septal velocities of mitral annulus; E/e’: Ratio of early diastolic mitral inflow to early diastolic mitral annular velocity; IVS: Thickness of ventricular septum; LVPW: Thickness of left ventricular posterior wall; SBP: Systolic blood pressure; DBP: Diastolic pressure; HR: Heart rate.

SCFP	β-blocker (n=11)	β-blocker+CCB±nitrate (n=5)	β-blocker+nitrate (n=5)	P-value
Depic, mm	3.4±0.8	4.0±1.1	4.2±1.5	NS
cTFC, frames	33.7±13.4	45.2±12.3	30.8±3.3	NS
E, cm/s	73.6±8.0	67.4±22	85.3±63.3	NS
A, cm/s	52.6±17.5	67.8±28.9	53±23.6	NS
DT, sec	191±52.2	206±23	177.5±14.4	NS
E/A	1.3±0.9	0.88±0.16	1.3±1.1	NS
e’_L _,cm/s	8.8±2.3	7.8±0.8	9.1±2.8	NS
e’s, cm/s	8.7±2.2	7±1.9	8.8±2.1	NS
E/e’	8.2±2.4	8.8±1.7	7.3±2.5	NS
Myocardial mass, gr/m^2^	90.7±14.9	95±10	82±10.4	NS
SBP, mmHg	132.1±18.7	133.8±13.8	126.7±9.8	NS
DBP, mmHg	82±10.8	72.5±12.6	76.2±23.6	NS
HR, beat/min	70.2±6.3	65.8±7.8	76.2±23.6	NS

**Table 8:** Vasoactive therapy and indices of diastolic function in patients with left ventricular hypertrophy: delayed and normal epicardial coronary flow. LVH: Patients with left ventricular hypertrophy; Depic: Epicardial coronary diameter; cTFC: Corrected TIMI frame count; E-wave and A-wave: Peak early and late rates of diastolic left ventricular filling; DT: Time of decrease in early diastolic left ventricular filling; e'_L_ and e'_S_: Early diastolic velocities of left ventricular wall and septum; E/e’: Index of left ventricular preload; IVS: Thickness of ventricular septum; LVPW: Thickness of left ventricular posterior wall; SBP: Systolic blood pressure; DBP: Diastolic pressure; HR: Heart rate.

LVH	β-blocker (n=10)	β-blocker+CCB±nitrate (n=12)	β-blocker+nitrate (n=5)	P-value
Depic, mm	3.8±0.9	3.6±0.9	3.6±0.6	NS
cTFC, frames	24.2±6.7	30.1±16.3	30.4±9.3	NS
E, cm/s	63.7±12.1	59.1±11.3	62.4±16.2	NS
A, cm/s	54.9±16.2	55.3±23.7	57.4±22.0	NS
DT, sec	177.2±33	183.2±37.3	186±30.3	NS
E/A	1.3±0.8	1.7±1.6	1.4±1.2	NS
e’_L_,cm/s	8.6±2.0	8.2±1.8	6.3±1.8	NS
e’s, cm/s	7.5±1.5	7.9±1.5	6±1.4	NS
E/e’	9.0±2.5	7.7±1.6	10.4±3.7	NS
Myocardial mass, gr/m^2^	124.7±23	127.3±18.8	150±25.3	NS
SBP, mmHg	132±16.4	130.2±3.9	147.4±17.1	NS
DBP, mmHg	85±4.1	82.5 ± 5.0	88.2 ± 28.9	NS
HR, beat/min	74.2±8.7	69.4±4.7	68±5.0	NS

## Discussion

Our results add to the current knowledge-specific data concerning the difference in diastolic function and the correlates of E/e’ between two patient groups without obstructive coronary disease: SCFP and patients with hypertension, ventricular hypertrophy, and variably delayed epicardial coronary flow.

The two major determinants of left ventricular filling are ventricular relaxation and effective chamber compliance. Ventricular relaxation is a complex energy-dependent process during which the contractile elements are deactivated and return to their precontraction length [14]. The relaxation in the heart is dependent on the left ventricular load and the uniformity of myocyte inactivation. The chamber compliance describes the passive properties of the left ventricle during the diastolic flow of blood across the mitral valve [14]. A decrease in chamber compliance can be caused by increased myocardial stiffness (viscoelastic properties of the myocardium) or an increase in LV volume. A decrease in chamber compliance will increase left ventricular filling pressure [14]. For patients with preserved systolic function, the ratio of early mitral inflow velocity to early diastolic velocity of the mitral annulus (E/e’) evaluated at echocardiography correlates better with the mean LV diastolic pressures than any other Doppler variable [15]. Moreover, E/e’ <8 accurately predicts normal mean LV diastolic pressure, and E/e’>15 identifies increased mean LV diastolic pressure. Wide variability in diastolic pressure has been reported in subjects with an E/e’ of 8 to 15 [16].

The mitral annular velocity (e′) was found to be weakly correlated in studies with invasive indices of relaxation (e.g., the constant of relaxation) [15]. However, е‘ could be used as a reliable index of diastolic function in patients with established heart disease because the effect of preload on e’ decreases with worsening relaxation [16]. In disease states, relaxation abnormalities occur early and often precede significant contractile dysfunction [13]. The slow coronary flow phenomenon is a well-recognized pathology characterized by impaired epicardial coronary flow in the absence of any myocardial abnormality or coronary stenosis of >30% [17]. It has been previously found that despite a reduction in the longitudinal function in SCFP during speckle tracking echocardiography, the conventional echocardiographic tissue Doppler parameters of biventricular diastolic function did not differ between SCFP and normal subjects [18].

According to earlier studies, the epicardial coronary flow in cases with left ventricular hypertrophy was frequently characterized as being delayed at angiography [19], which is similar to the epicardial flow of patients with SCFP. Our analysis showed an abnormal diversion in diastolic function parameters (e’ and E/e’) in patients with hypertensive heart disease and weakly or moderately elevated LV mass compared to those diagnosed with SCFP. A few studies emphasized that the coronary flow in hypertension was substantially reduced when adjusted for LV mass [20] and was inversely correlated with the volume of myocardial mass and myocardial fibrosis [21]. The tendency toward abnormal perfusion at rest in the hypertrophied heart has been related to arterial remodeling that is out of proportion to the hypertrophied myocardium and the increased extravascular forces acting on intramural arteries [21,22]. Notably, the index of left ventricular mass in diastole could accurately predict the invasively measured left ventricular pressure and E/e’ ratio in cohorts with hypertensive heart disease [23].

It has been recently reported that the epicardial coronary arterial lumen volume to LV mass ratio modified the coronary arterial resistance in the presence of only minimal coronary disease [24]. Our results replicate the findings on coronary flow in the setting of hypertensive heart disease and extend the knowledge in the non-obstructive coronary disease field by comparing it with SCFP. Furthermore, we demonstrated that delayed epicardial flow in left ventricular hypertrophy is related to more severely reduced left ventricular relaxation and elevated LV filling pressures compared to SCFP.

In agreement with previous findings [7,25,26], our results also suggested that the long-term usage of β-blockers and calcium antagonists could not normalize the elevated left ventricular filling pressures in patients with left ventricular hypertrophy, despite their beneficial effects on diastolic parameters and myocardial mass. Conversely, long-term therapy with oral nitrates may predispose patients to higher LV diastolic pressures via different mechanisms [27,28].

The cases of uncertain prognostic accuracy of E/e’ (normal subjects, heavy mitral annular calcification, mitral valve disease, obstructive coronary disease, and regional ventricular dysfunction) [13] were excluded from the study; thus, an increased E/e’ ratio is expected to reliably correlate with higher LV filling pressures in our cohort. The e’ decreases with age [29]. On average, our study patients were younger (mean age: 57.3±8.7 years; two-thirds of patients being younger than 60 years); thus, the observed abnormalities in the diastolic parameters e’ and E/e’ should be discussed in relation to their cardiovascular morbidities.

## Conclusions

Patients with microvascular angina represent a diverse population. The echocardiographic parameters of left ventricular relaxation (e’) and end-diastolic pressure (E/e’) are abnormally altered in the population with left ventricular hypertrophy compared to those in SCFP. The delayed epicardial flow further impairs diastolic function in hypertensive patients with hypertrophy and non-obstructive coronary disease.
